# Promotion of nitric oxide production: mechanisms, strategies, and possibilities

**DOI:** 10.3389/fphys.2025.1545044

**Published:** 2025-01-23

**Authors:** Marcos Gonzalez, Sarah Clayton, Eric Wauson, Daniel Christian, Quang-Kim Tran

**Affiliations:** Department of Physiology and Pharmacology, Des Moines University Medicine and Health Sciences, West Des Moines, IA, United States

**Keywords:** nitric oxide, eNOS, endothelium, preclinical evidence, clinical trials

## Abstract

The discovery of nitric oxide (NO) and the role of endothelial cells (ECs) in its production has revolutionized medicine. NO can be produced by isoforms of NO synthases (NOS), including the neuronal (nNOS), inducible (iNOS), and endothelial isoforms (eNOS), and via the non-classical nitrate-nitrite-NO pathway. In particular, endothelium-derived NO, produced by eNOS, is essential for cardiovascular health. Endothelium-derived NO activates soluble guanylate cyclase (sGC) in vascular smooth muscle cells (VSMCs), elevating cyclic GMP (cGMP), causing vasodilation. Over the past four decades, the importance of this pathway in cardiovascular health has fueled the search for strategies to enhance NO bioavailability and/or preserve the outcomes of NO’s actions. Currently approved approaches operate in three directions: 1) providing exogenous NO, 2) promoting sGC activity, and 3) preventing degradation of cGMP by inhibiting phosphodiesterase 5 activity. Despite clear benefits, these approaches face challenges such as the development of nitrate tolerance and endothelial dysfunction. This highlights the need for sustainable options that promote endogenous NO production. This review will focus on strategies to promote endogenous NO production. A detailed review of the mechanisms regulating eNOS activity will be first provided, followed by a review of strategies to promote endogenous NO production based on the levels of available preclinical and clinical evidence, and perspectives on future possibilities.

## Nitric oxide bioavailability and the need to promote its endogenous production

### NO synthases and general effects of NO

NO-containing compounds have been used in medicine for over 160 years; however, it was not until the early 1980s that NO was discovered as the ingredient that exerts the therapeutic effects and ECs as the key source of vascular NO (Katsuki et al., 1977; Arnold et al., 1977; Furchgott and Zawadzki, 1980; Ignarro et al., 1987; Palmer et al., 1987). We now know NO can be produced by three NO synthases and via nitrate-nitrite-NO conversion (Bredt et al., 1991; Janssens et al., 1992; Geller et al., 1993; Benjamin et al., 1994; Lundberg et al., 1994; Stuehr, 1997). The NO synthases are classified a neuronal NOS (encoded by *NOS1*), inducible NOS (encoded by *NOS2*), and eNOS (encoded by *NOS3*). Among these sources, endothelium-derived NO plays a vital role in regulating vascular tone, inhibiting inflammation, and preventing thrombosis (Alheid et al., 1987; Forstermann et al., 1989; Bredt and Snyder, 1990; Mitchell et al., 1991; Forstermann et al., 1991). Endothelium-derived NO diffuses into the circulation and the underlying VSMCs, where it activates sGC, which enhances cGMP, causing vasodilation (Arnold et al., 1977; Ignarro et al., 1986a; Ignarro et al., 1986b; Forstermann et al., 1994). The critical role of eNOS in controlling vascular tone was documented by findings that pharmacological inhibition of NOS causes hypertension (Rees et al., 1989) and deletion of *NOS3* results in high blood pressure (Huang et al., 1995). Beyond activating sGC and enhancing cGMP, NO exerts numerous other effects. For example, endothelium-derived NO directly suppresses the electrical excitability of VSMCs by inhibiting the action of T-type and L-type voltage-gated Ca^2+^ channels in small arteries, and reduced NO availability can trigger transient depolarization in normally quiescent VSMCs leading to vasospasm (Smith et al., 2020). Actions of NO leading to smooth muscle relaxation are important for cardiovascular, respiratory, renal and digestive functions. NO is also critical in brain function as a neurotransmitter and immune responses.

### Efforts to increase NO bioavailability

Dysfunction and uncoupling of eNOS are associated with cardiovascular diseases (CVD) (Janaszak-Jasiecka et al., 2023; Heitzer et al., 2000a; Munzel et al., 2005), and increased eNOS expression and reversal of eNOS uncoupling in experimental models improves vascular function (Li et al., 2006). Approaches to increase NO bioavailability have been intensively researched. Some studies suggest that diets that are high in *antioxidants or antioxidant supplementation* can help preserve vascular health and prevent CVD by reducing oxidative stress and improving endothelial function. However, a blanket recommendation has not been made in clinical guidelines as there are needs for stronger evidence and determinations of effective doses and specific patient populations that might benefit (Varadharaj et al., 2017). *Dietary nitrates and nitrites*, found in foods such as beetroot and leafy greens, can be converted to NO and have shown promising results in improving vascular function and lowering blood pressure. However, these are not yet recommended in clinical guidelines for prevention or management of CVD (Blekkenhorst et al., 2018). Current options in clinical practice guidelines focus mainly on downstream components of NO signaling, such as NO inhalation, use of NO donors, sGC stimulators/activators, or inhibition of phosphodiesterase (PDE) 5 (Chen et al., 2002; Rapoport et al., 1987; Bonderman et al., 2014; Ignarro et al., 1982; Kraehling and Sessa, 2017; Lundberg et al., 2015). However, challenges such as short-lived effects and the development of nitrate tolerance and endothelial dysfunction have limited their efficacy (Rapoport et al., 1987; Munzel et al., 1995a; Munzel et al., 1995b; Erdmann et al., 2013; Knorr et al., 2011; Munzel et al., 2014; Munzel et al., 2013; Oelze et al., 2013). There is thus a strong need to develop new and improve existing approaches to promote the endogenous production of NO.

## The regulation of eNOS

**FIGURE 1:**
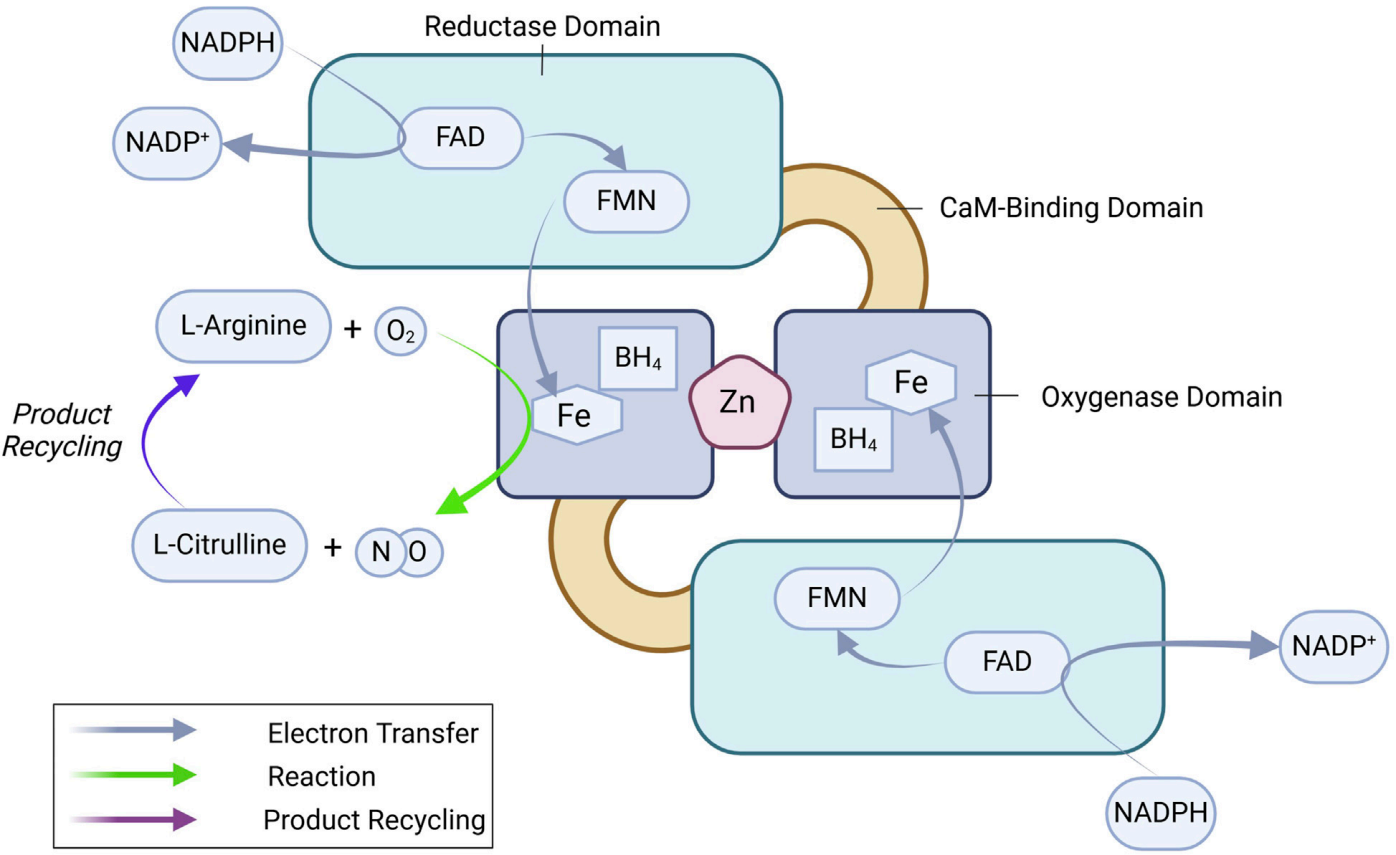
eNOS dimer and key enzymatic reactions leading to NO production. See text for details. BH_4_, tetrahydrobiopterin; FAD, flavin adenine dinucleotide; FMN, flavin mononucleotide; NADPH, nicotinamide adenine dinucleotide phosphate.

eNOS is a ∼133 kDa homo-dimeric oxidoreductase enzyme with an N-terminal oxygenase domain that binds L-arginine and tetrahydrobiopterin (BH_4_) and a C-terminal reductase domain that transfers electrons from nicotinamide adenine dinucleotide phosphate (NADPH) via flavin adenine dinucleotide (FAD) and flavin mononucleotide (FMN) (Figure 1) (Stuehr, 1997; Palmer et al., 1988; Stuehr et al., 2005; Marsden et al., 1992). Dimerization is essential for eNOS function, stabilizing the enzyme and ensuring efficient electron transfer (List et al., 1997). Ca^2+^-bound calmodulin (CaM) binds to the CaM-binding domain and initiates electron flow from the reductase domain to the oxygenase domain, where NO is synthesized from L-arginine (Busse and Mulsch, 1990; Venema et al., 1996). eNOS-mediated production of NO follows a two-step process: 1) hydroxylation of L-arginine to Nω-hydroxy-L-arginine, and 2) further oxidation to generate L-citrulline and NO. Tetrahydrobiopterin (BH_4_) plays a vital role in this reaction by stabilizing the eNOS dimer and preventing the formation of superoxide, a potentially harmful byproduct, ensuring NO synthesis proceeds efficiently (Xia et al., 1998a; Xia et al., 1998b). eNOS regulation is a highly complex process, integrating multiple layers of control to fine-tune NO production according to physiological needs. These layers include transcriptional regulation, post-translational modifications, and protein-protein interactions.

### Transcriptional regulation of eNOS

**FIGURE 2:**
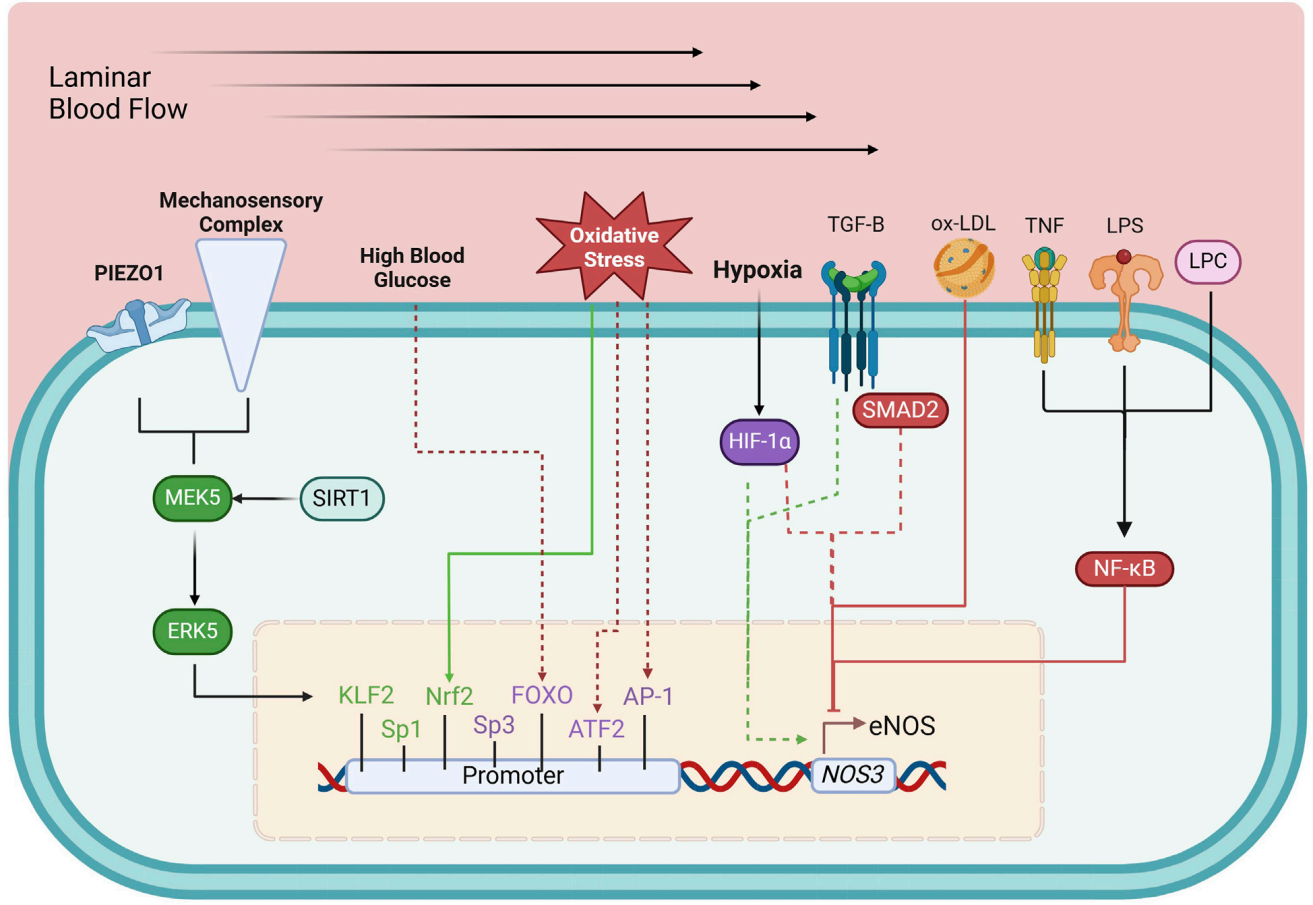
Transcriptional regulation of eNOS. Green boxes, upregulating factors; purple boxes, dual-regulating factors; red boxes, down-regulating factors; solid arrows, connection between environmental and metabolic elements to their respective regulating factors; dashed arrows, circumstantial elements that determine the effects of dual-regulating factors; AP-1, activator protein 1; ATF-2, activating transcription factor 2; ERK5, extracellular-regulated kinase 5; FOXO, forkhead box O; HIF-1α, hypoxia-inducible factor 1-α; KLF2, Krüppel-like factor 2; LPC, lysophosphatidylcholine; LPS, lipopolysaccharide; MEK5, Mitogen-activated protein kinase kinase 5; Nrf2, Nuclear factor erythroid 2-related factor; NF-κB, Nuclear Factor kappa-light-chain-enhancer of activated B cells; ox-LDL, oxidized low-density lipoprotein; SIRT1, sirtuin 1; SMAD2, mothers against decapentaplegic homolog-2; Sp1, specificity protein 1; Sp3, specificity protein 3; TGF-β, tumor growth factor β; TNF, tumor necrosis factor. See text for details.

The *NOS3* gene is located on chromosome 7 (7q35-36) and is regulated by a promoter region containing binding sites for multiple transcription factors, including Krüppel-like Factor 2 (KLF2), Specificity protein 1 (Sp1), Specificity protein 3 (Sp3), Ets1, mothers against decapentaplegic homolog-2 (Smad2), and Nuclear factor erythroid 2-related factor (Nrf2), among others (Karantzoulis-Fegaras et al., 1999; Laumonnier et al., 2000; Balligand et al., 2009; Fish and Marsden, 2006). These factors dynamically regulate eNOS expression in response to physiological signals. Initially thought to be constitutively expressed, *NOS3* is now recognized to be highly responsive to various regulatory stimuli, which adjust its transcription to match the vascular environment’s needs (Balligand et al., 2009; Black et al., 1998; Mattsson et al., 1997; Nadaud et al., 1996; Nishida et al., 1992; Ou et al., 2005; Searles, 2006). Transcriptional regulation of eNOS can be categorized into upregulating, downregulating, and dual-regulating factors (Figure 2).

#### Upregulating factors

*Shear stress*, the mechanical stimulus exerted on the endothelium by laminar blood flow, upregulates eNOS expression (Nadaud et al., 1996) by triggering PIEZO1 Ca^2+^ channels (Wang et al., 2016) and a mechanosensory complex leading to the activation of *KLF2*, which binds directly to the *NOS3* promoter (Wang et al., 2010; Lin et al., 2005). *Sp1* regulates basal *NOS3* expression and responds to stimuli like growth factors and hypoxia, enhancing NO production to maintain vascular tone (Tang et al., 1995; Wariishi et al., 1995). Additionally, *Nrf2*, activated by oxidative stress, enhances *NOS3* transcription by upregulating antioxidant response elements in the *NOS3* promoter (Wu et al., 2019).

#### Downregulating factors

NF-κB (Nuclear Factor kappa-light-chain-enhancer of activated B cells) represses eNOS expression, particularly in inflammation. NF-κB is activated by pro-inflammatory stimuli such as tumor necrosis factor-alpha (TNF-α), lipopolysaccharide (LPS), and oxidized low-density lipoprotein (ox-LDL). These molecules trigger signaling events that lead to NF-κB translocation into the nucleus, where it inhibits eNOS transcription (Nishida et al., 1992; Neumann et al., 2004). LPS and ox-LDL, both associated with oxidative stress and vascular inflammation, promote NF-κB activation, further repressing eNOS expression (Liao et al., 1995; Lu et al., 1996).

#### Dual-regulating factors

Certain factors can both up- or downregulate eNOS expression depending on cellular conditions. Tumor growth factor β (TGF-β)/Smad2 can enhance eNOS transcription in a healthy endothelium but suppress it in chronic inflammation or vascular injury (Saura et al., 2002). Hypoxia-inducible factor-1α (HIF-1α) also exhibits dual regulation: during acute hypoxia, it stimulates eNOS expression to ensure adequate NO production but may suppress eNOS in chronic hypoxia, causing maladaptive vascular changes (McQuillan et al., 1994; Fish et al., 2010).

*Post-transcriptional mechanisms* offer additional precision by modulating the stability and translation of eNOS mRNA. Elements such as miRNAs (e.g., miR-92a) and long non-coding RNAs (lncRNAs) can either enhance or suppress NO production in response to physiological conditions (Lin et al., 2005; Miao et al., 2018; Suarez et al., 2007; Man et al., 2018; Bonauer et al., 2009).

### Post-translational modifications (PTMs) of eNOS

**FIGURE 3:**
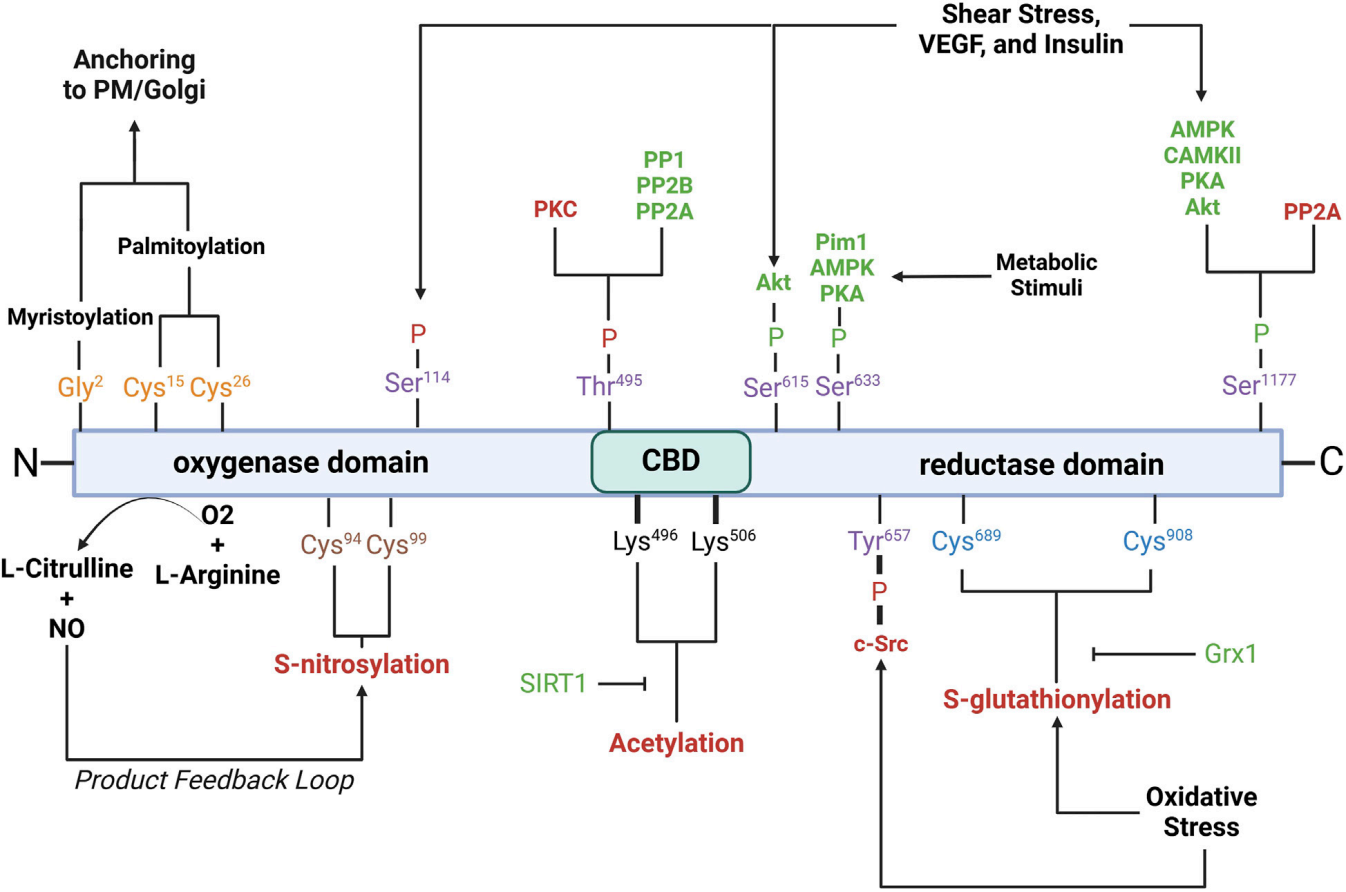
Schematic of eNOS domains with key residues involved in post-translational modifications. Purple residues, phosphorylation sites; yellow residues, myristoylation/palmitoylation sites; brown residues, S-nitrosylation sites; black residues, acetylation sites; blue residues, S-glutathionylation sites. Red- and green-letter enzymes, inhibitory and stimulatory effects on eNOS, respectively. Akt, protein kinase B; AMPK, AMP-activated protein kinase; CaMKII, calcium/calmodulin-dependent protein kinase II; c-Src, cellular sarcoma; Grx1, glutaredoxin; Pim1, proviral integration site for Moloney murine leukemia virus 1; PKA, protein kinase A; PKC, protein kinase C; PP1, protein phosphatase 1; PP2A, protein phosphatase 2a; PP2B, protein phosphatase 2b; PM, plasma membrane; SIRT1, sirtuin 1; VEGF, vascular endothelial growth factor. See text for further details.

eNOS activity is also intricately regulated by various PTMs, including phosphorylation, acetylation, S-nitrosylation, and palmitoylation. These modifications play critical roles in modulating eNOS enzymatic activity, localization, and interactions with other cellular components (Figure 3).

*Phosphorylation* is a critical modification that modulates eNOS activity and is tightly regulated by various kinases and phosphatases in response to physiological cues such as shear stress, hypoxia, and growth factors. *Serine 1177* is located on the C-terminal reductase domain and when phosphorylated, enhances eNOS activity by facilitating electron flow from NADPH to the heme domain, contributing to NO production (Fulton et al., 1999; Scotland et al., 2002; Kashiwagi et al., 2013; Li Q. et al., 2013; Park et al., 2016). Ser1177 phosphorylation is key positive regulator of eNOS function (Dimmeler et al., 1999; Tomada et al., 2014). Stimuli such as shear stress, vascular endothelial growth factor (VEGF), and insulin activate kinases including protein kinase B (Akt), AMP-activated protein kinase (AMPK), calcium/calmodulin-dependent protein kinase II (CaMKII), protein kinase A (PKA), and protein kinase G (PKG), which phosphorylate Ser1177 (Fulton et al., 1999; Dimmeler et al., 1999; Michell et al., 1999; Chen et al., 1999; Fleming et al., 2001; Atochin et al., 2007). Phosphorylation by Akt, in particular, is essential for eNOS activation in endothelial cells in response to VEGF and shear stress (Dimmeler et al., 1999; Di Lorenzo et al., 2013). Ser1177 phosphorylation also increases the Ca^2+^ sensitivity of the synthase, permitting CaM binding and enzyme activation at lower intracellular Ca^2+^ levels (Tran et al., 2009; Mount et al., 2007; McCabe et al., 2000). *Serine 633* is phosphorylated in response to shear stress, exercise, and metabolic stimuli (Mount et al., 2007). Ser633 phosphorylation by PKA and AMPK during physical activity improves NO bioavailability and supports vascular homeostasis (Mount et al., 2007; Michell et al., 2002). *Serine 615* phosphorylation enhances eNOS activity, working cooperatively with Ser1177 to enhance the binding affinity of the Ca^2^⁺-CaM complex to eNOS, a critical step for eNOS activation, which ensures a robust response to Ca^2^⁺ signals (Tran et al., 2009; Bauer et al., 2003). *Threonine 495* phosphorylation, in contrast, inhibits eNOS by suppressing CaM binding (Fleming et al., 2001). Kinases like AMPK and PKC mediate this modification, especially during oxidative stress (Chen et al., 1999). Agonists such as bradykinin promote NO release by inducing Thr495 dephosphorylation, allowing CaM to activate eNOS (Harris et al., 2001). This dephosphorylation is mediated by calcineurin and inhibited by cyclosporine A (Harris et al., 2001). The balance between Thr495 phosphorylation and dephosphorylation is crucial for regulating eNOS activity and NO production. *Serine 114* is phosphorylated in response to shear stress (Mount et al., 2007; Gallis et al., 1999). Though its role remains debatable, phosphorylation at Ser114 is considered a negative regulator of eNOS activity (Mount et al., 2007), supported by the observations that its dephosphorylation by VEGF treatment enhances eNOS function (Bauer et al., 2003) and that a phospho-null mutation here inhibits eNOS activity (Kou et al., 2002; Li et al., 2007).

*Acetylation* regulates eNOS interactions with other proteins, its plasma membrane localization, and overall enzymatic efficiency. Acetylation at *lysine 609* affects eNOS interaction with heat shock protein 90 (Hsp90) and CaM, both essential for eNOS activation (Taubert et al., 2004). Lysine 609 acetylation is mediated by histone deacetylase 3 and inhibits eNOS activity by preventing proper electron transfer (Jung et al., 2010). In contrast, its deacetylation by sirtuin 1 (SIRT1) restores eNOS activity, enhancing eNOS-CaM interaction (Donato et al., 2011; Arunachalam et al., 2010).

*S-nitrosylation* is a reversible modification that constrains NO synthesis via a product feedback mechanism (Lipton et al., 1993; Erwin et al., 2005; Lima et al., 2010). S-nitrosylation involves the covalent attachment of a NO group to cysteine thiols, specifically Cys94 and Cys99 of eNOS, forming S-nitrosothiols (SNOs) (Erwin et al., 2006). Cys94 and Cys99 are located within the zinc tetrathiolate cluster (Erwin et al., 2005) that is important for the eNOS dimer interface; nevertheless, mutation of these sites does not disrupt dimer formation (Erwin et al., 2005). Paradoxically, agonist stimulation, which increases NO production, also promotes rapid denitrosylation of eNOS, in a similar timeframe as phosphorylation at Ser1177 (Erwin et al., 2005). S-nitrosylated eNOS exhibits reduced catalytic activity, which can be reversed with the release of NO. The subcellular localization of eNOS influences the degree of S-nitrosylation, with membrane-bound eNOS being more heavily nitrosylated than its cytosolic counterpart due to higher NO production at the membrane (Erwin et al., 2006).

*Glutathionylation* is a reversible post-translational modification in which the tripeptide glutathione attaches to cysteine residues in eNOS, notably Cys689 and Cys908, in the reductase domain (Chen et al., 2010). Glutathionylation is promoted by oxidative stress and results in decreased NO production and increased superoxide generation due to disrupted flavin-dependent electron transport (Chen et al., 2010; Crabtree et al., 2013). Fortunately, this modification is reversible through the action of glutaredoxin (Grx1), which interacts closely with eNOS (Chen et al., 2013). Loss of Grx1, either by oxidative stress or genetic silencing, increases eNOS glutathionylation and further NO synthesis (Chen et al., 2013).

*Palmitoylation and myristoylation* are lipid modifications that regulate the localization and activity of eNOS. Myristoylation, the irreversible attachment of myristic acid to Gly2, anchors eNOS to membranes such as the plasma membrane and Golgi apparatus (Liu et al., 1995; Sessa et al., 1995). Myristoylation is a prerequisite for palmitoylation, a reversible process where palmitic acid binds to Cys15 and Cys26, anchoring eNOS within plasmalemmal caveolae (Fernandez-Hernando et al., 2006). The reversible cycle of palmitoylation and depalmitoylation allows eNOS to dynamically shift between membrane locations in response to physiological signals (Yeh et al., 1999).

### Regulation of eNOS by protein-protein interactions (PPIs)

**FIGURE 4:**
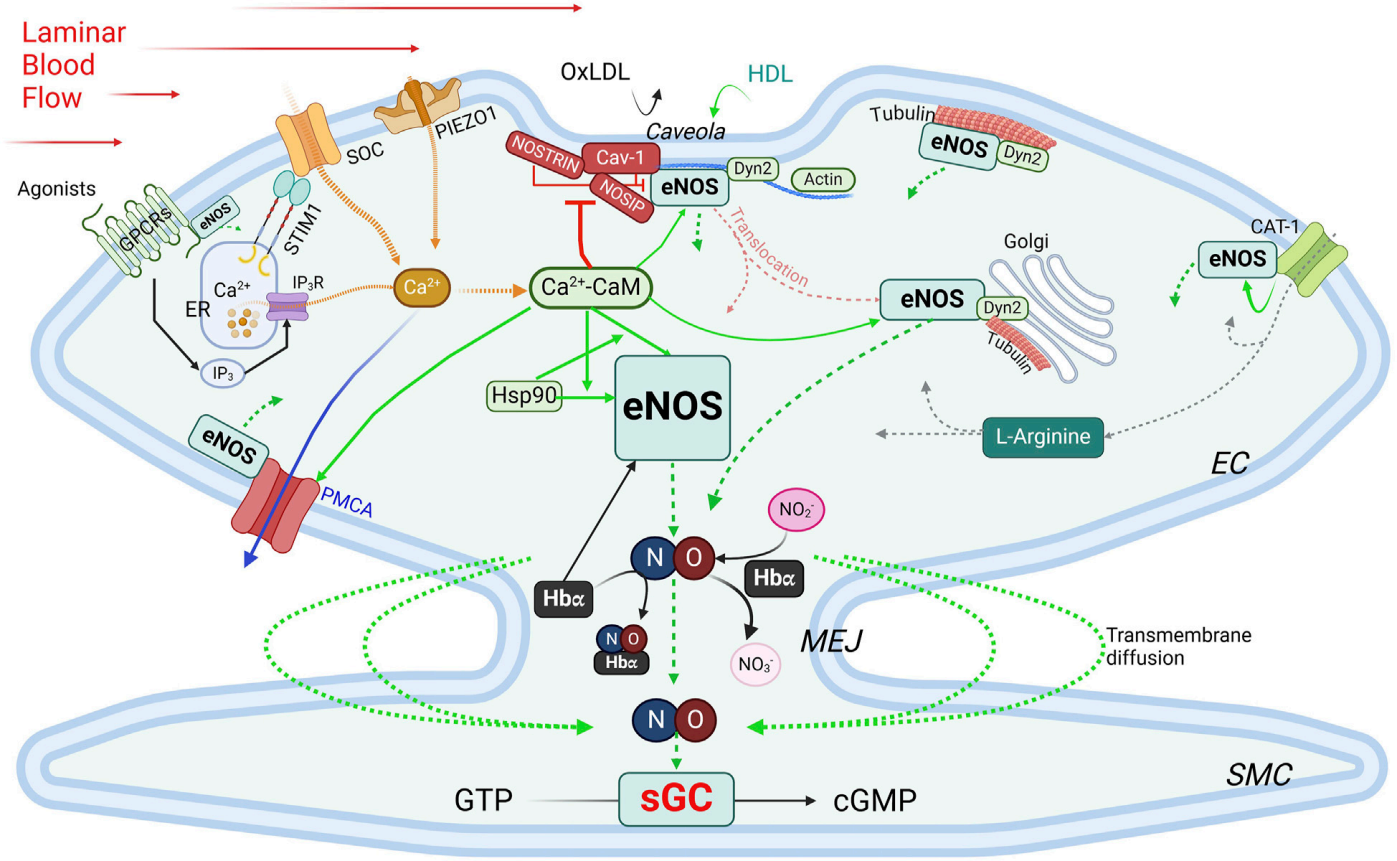
Regulation of eNOS by PPIs. Green boxes, stimulatory interacting partners; red boxes, inhibitory interacting partners. Green arrows indicate stimulatory interactions; dotted green arrows, NO production/diffusion; yellow dotted arrows, increase of intracellular Ca^2+^; solid blue arrow, Ca^2+^ extrusion; grey dotted arrows, L-Arginine transportation. See text for details. CAT-1, cationic anion transporter 1; Cav-1, caveolin-1; Ca^2+^-CaM, calcium-bound calmodulin; Dyn2, dynamin-2; EC, endothelial cells; ER, endoplasmic reticulum; GPCR, G protein-coupled receptor; Hbα, α subunit of hemoglobin; HDL, high-density lipoprotein; Hsp90, heat shock protein 90; IP_3_, inositol trisphosphate; IP_3_R, inositol trisphosphate receptor; MEJ, myoendothelial junctions; NOSIP, eNOS-interacting protein; NOSTRIN, eNOS trafficking inducer; ox-LDL, oxidized low-density lipoprotein; PMCA, plasma membrane Ca^2+^-ATPase; SMC, smooth muscle cell; SOC, store-operated Ca^2+^ channel; STIM1, stromal interaction protein 1.

eNOS activity is intricately regulated through its interactions with various binding partners. These interactions play essential roles in modulating its localization, dimerization, and activation, ensuring that eNOS responds appropriately to cellular and environmental signals (Figure 4).

*Caveolin-1* (Cav-1) is the main caveolin of caveolae in endothelial cells (Feron et al., 1996; Garcia-Cardena et al., 1996; Ju et al., 1997). Cav-1 binds to eNOS at the caveolin-scaffolding domain (CSD, a.a. 81–101), preventing eNOS interaction with activators, thus inhibiting NO production under basal conditions (Garcia-Cardena et al., 1997; Michel et al., 1997). Physiological stimuli such as shear stress or G protein-coupled receptor (GPCR) agonists like bradykinin promote Ca^2+^ entry, leading to dissociation of the Cav-1-eNOS complex and allowing eNOS to be activated via phosphorylation by kinases such as Akt (Feron et al., 1998). Cav-1 deletion enhances endothelium-dependent relaxation and lowers blood pressure (Murata et al., 2007; Razani et al., 2001). Interaction with Cav-1, however, avoids excessive or aberrant eNOS activity, and Cav-1 deficiency can cause pulmonary hypertension and cardiomyopathy (Drab et al., 2001; Zhao et al., 2002). While plasma membrane localization is ideal for eNOS activation, this also exposes eNOS to external factors like ox-LDL and high-density lipoprotein (HDL). Ox-LDL can disrupt the cholesterol-rich environment in caveolae, thereby reducing NO production specifically from plasma membrane-bound eNOS, whereas Golgi-localized eNOS is more resistant (Zhang et al., 2006; Blair et al., 1999). HDL, on the other hand, provides cholesterol esters to maintain caveolae’s cholesterol, and via palmitoylation, retain eNOS at the plasma membrane (Uittenbogaard et al., 2000).

*Ca^2+^-calmodulin* eNOS activity is regulated by a complex and tightly regulated network of functionally and physically interacting proteins involved in Ca^2+^ signaling, including Ca^2+^ entry, the Ca^2+^ sensor CaM, and Ca^2+^ efflux. CaM, in its Ca^2^⁺-bound form (Ca^2+^-CaM), is required for activating eNOS by facilitating electron flow from the reductase domain to the oxygenase domain and promoting dimerization of the latter (Forstermann et al., 1991; Busse and Mulsch, 1990; Hellermann and Solomonson, 1997). The Ca^2+^ entry mechanisms in ECs play important roles in this process, as evidenced by the observations that removal of extracellular Ca^2+^ or inhibition of CaM suppresses agonist-induced NO production (Forstermann et al., 1991; Busse and Mulsch, 1990; Singer and Peach, 1982). *Two major Ca* ^*2+*^ *entry pathways* are important for NO production: store-operated Ca^2+^ entry (SOCE), the main agonist-induced Ca^2+^ entry mechanism in ECs (Abdullaev et al., 2008; Tran, 2020), and mechanosensitive Ca^2+^ entry, stimulated by blood shear stress. For activation of SOCE, the stromal interaction molecule 1 (STIM1) is required (Roos et al., 2005; Soboloff et al., 2012). Vascular STIM1 plays opposing roles in the regulation of vascular tone; smooth muscle cell STIM1 is important for VSMC contractility, proliferation and the development of hypertension (Kassan et al., 2016). On the other hand, endothelial STIM1 plays a critical role in the activation of eNOS to produce NO, such that EC-specific deletion of the *STIM1* gene impairs endothelium-dependent vasorelaxation and increases blood pressure (Nishimoto et al., 2018). Shear stress, a potent physiological stimulus of NO production, stimulates PIEZO1 mechanosensitive channel for Ca^2+^ entry (Wang et al., 2016; Ranade et al., 2014; Li J. et al., 2014). Once bound to Ca^2+^, CaM regulates eNOS activity via *two important mechanisms*. First, the Ca^2+^-CaM complex displaces eNOS from the inhibitory interaction with caveolin (Michel et al., 1997). Second, CaM binds eNOS at a canonical CaM-binding site encompassing amino acids 493–512 (Venema et al., 1996) with a K_*d*_ value of ∼0.2 nM (Tran et al., 2005). This interaction is inhibited at low Ca^2+^ concentration by the autoinhibitory domain (residues 595–639) (Chen and Wu, 2000). Upon increases in intracellular Ca^2+^, Ca^2+^-bound CaM binds eNOS, displacing the autoinhibitory loop and facilitating electron transfer between the two domains (Pollock et al., 1991; Nishida and Ortiz de Montellano, 1999). Phosphorylation at Ser615 within this loop reduces the concentration of Ca^2+^ required for eNOS-CaM interaction, alleviating the autoinhibitory effect (Tran et al., 2008). CaM binding also enhances eNOS phosphorylation at Ser1177, which, in combination with Ser615 phosphorylation, further increases the Ca^2+^ sensitivity of eNOS-CaM interaction and synthase activation (Fleming et al., 2001; Tran et al., 2009; Tsukahara et al., 1994; Fleming et al., 1997). These phosphorylation events facilitate significant eNOS-CaM interaction and synthase activity at basal level of intracellular Ca^2+^ and explain the effects of factors that promote NO production without triggering significant increases in global cytoplasmic Ca^2+^. *Interaction with Ca* ^*2+*^ *efflux channel* – The plasma membrane Ca^2+^-ATPase (PMCA) is a key Ca^2+^ extrusion mechanism in ECs (Wang et al., 2002; Tran et al., 2003). eNOS directly interacts via residues 735 – 934 in its reductase domain with residues 428–651 in the catalytic domain of PMCA (Holton et al., 2010). This interaction enhances phosphorylation of Thr495 in the CaM-binding domain of eNOS (Holton et al., 2010), which reduces eNOS-CaM interaction (Fleming et al., 2001). The Ca^2+^/CaM-dependent phosphatase calcineurin is associated with the PMCA-eNOS complex, suggesting a potential role in the effect of PMCA expression on Thr495 phosphorylation status (Holton et al., 2010). Interestingly, PMCA activity is controlled by CaM interaction (Di Leva et al., 2008) and Ca^2+^ extrusion via PMCA moderates eNOS-CaM binding and synthase activation (Tran et al., 2016). Thus, CaM binding fine-tunes NO synthesis in response to subtle changes in the Ca^2+^ concentration surrounding eNOS and by control the activities of many of its interacting partners. With sub-nanomolar affinity for its interaction with CaM and limited abundance of CaM in ECs, eNOS expression level and activation in turn significantly influences the CaM-binding proteome in ECs (Tran et al., 2005; Tran et al., 2003). Treatment with 17β-estradiol or an agonist of the G protein-coupled estrogen receptor enhances CaM expression level substantially in ECs and promotes eNOS activity by moderating Ca^2+^ entry and efflux, enhancing eNOS-CaM interaction and associated eNOS phosphorylation (Tran, 2020; Tran et al., 2016; Fredette et al., 2017; Tran et al., 2015; Terry et al., 2017).

*Interactions with components of the cytoskeleton and membrane-targeted proteins* *Actin* interacts with an eight-amino acid motif (Jo et al., 2011; Higashi et al., 2002; Maier et al., 2000; Setoguchi et al., 2001; Fukuda et al., 2002; Holowatz and Kenney, 2011; Nystrom et al., 2004; Worthley et al., 2007) in the oxygenase domain of eNOS (Kondrikov et al., 2010). In *in vitro* assays, G-actin promotes eNOS activity more than does F actin (Su et al., 2003); however, a low G/F actin ratio appears to correlate with higher eNOS expression level (Searles et al., 2004). *Dynamin-2* (Dyn2) is a GTP-binding protein in the caveolae and the Golgi (McClure and Robinson, 1996). Dynamin-2 interacts directly with eNOS in these compartments in ECs; this interaction is enhanced by Ca^2+^ and increases eNOS activity (Cao et al., 2001). *NOSTRIN* (e*NOS tr*afficking *in*ducer), a 506-a.a. protein enriched in vascular tissues, interacts with eNOS via an SH3 domain, promotes eNOS redistribution from the membrane, and inhibits synthase activity (Zimmermann et al., 2002). It also interacts with a region spanning a.a. 1- 61 of cav-1, N-terminally from the cav-1 scaffolding domain, thus forming a ternary complex with eNOS and cav-1 (Schilling et al., 2006). NOSTRIN interacts with dynamin-2 and is required for recruitment of eNOS to dynamin-containing structures (Icking et al., 2005). *NOSIP* (e*NOS*-*i*nteracting *p*rotein) is another protein residing in caveolae that interacts with eNOS and promotes its translocation from the plasma membrane and inhibit NO production (Dedio et al., 2001). In addition, eNOS associates with *tubulin* (Dedio et al., 2001), which plays an important role in its trafficking to the Golgi. Acetylation of α tubulin is involved in stabilizing microtubules where eNOS is associated in the Golgi and is phosphorylated for basal activity (Giustiniani et al., 2009). *Interaction with GPCRs* – Evidence from *in vitro* studies indicates that eNOS can interact with the juxtamembranous regions of AT_1_R, ET_A_, ET_B_ and bradykinin B_2_ receptor (Marrero et al., 1999). While these interactions are likely important because activation of these receptors increases intracellular Ca^2+^, which is predicted to activate eNOS, they require further verification *in vivo*. Activity of eNOS is also regulated by its direct association with the *cationic amino acid transporter (CAT)-1*, the key transporter of L-arginine (Li et al., 2005). The promotion of eNOS activity by its interaction with CAT-1 is independent of L-arginine transport and is associated with enhanced phosphorylation at Ser1177 and Ser633 and reduced interaction with cav-1 (Li et al., 2005).

*Heat shock protein 90* (Hsp90) is a molecular chaperone that stabilizes eNOS, thereby promoting its activation. The substrate-binding region of Hsp90 binds to the oxygenase domain of eNOS between a.a. 310–323 (Fontana et al., 2002; Xu H. et al., 2007). This interaction is increased by stimuli such as VEGF, histamine, estrogen, and shear stress (Garcia-Cardena et al., 1998; Russell et al., 2000; Venema et al., 1997). Hsp90 binding enhances phosphorylation at Ser1177 by recruiting kinases such as Akt, which further boosts NO production (Fontana et al., 2002). Hsp90 is critical for eNOS activity, and is involved in a *reciprocal interactive relationship with eNOS and CaM*: Hsp90 enhances both the magnitude and sensitivity of eNOS activation in response to Ca^2+^-CaM; in turn, the effect of Hsp90 to promote eNOS activity is enhanced by Ca^2+^-CaM complex (Takahashi and Mendelsohn, 2003). Hsp90 also exerts a CaM-independent effect to promote eNOS activity (Takahashi and Mendelsohn, 2003).

*Hemoglobin alpha (Hbα)* The α, but not β, subunit of hemoglobin is expressed in ECs in resistance blood vessels, where it is concentrated in the myoendothelial junctions (MEJs) between ECs and VSMCs (Straub et al., 2012). Hbα regulates NO availability via three mechanisms. *First*, it can bind and release NO and thus can act as both a reservoir and scavenger of NO, thereby regulating NO-mediated processes (Straub et al., 2014a; Keller et al., 2022; Straub et al., 2014b). In its ferrous (Fe^2+^) form, Hbα binds NO with high affinity, yielding nitrate and ferric (Fe^3+^) Hbα, which binds NO with much lower affinity (Straub et al., 2012). The reduction of Fe^3+^ Hbα to Fe^2+^ Hbα, catalyzed by cytochrome b5 reductase 3 (CytB5R3), thus enhances the scavenging action of NO by Hbα (Straub et al., 2012). *Second*, Hbα associates at its residues 34–43 with the oxygenase domain of eNOS in a macromolecular complex (Straub et al., 2014a), an interaction dictated by amino acids ^36^SFPT^39^ in the Hbα sequence (Keller et al., 2022). However, excessive NO scavenging in conditions of elevated Hb concentrations in the MEJs can reduce NO bioavailability and induce endothelial dysfunction (Denton et al., 2021), increasing vascular resistance (Straub et al., 2014a). *Third*, endothelial Hbα can function as a nitrite reductase, which produces NO through reduction of nitrite in hypoxic condition (Keller et al., 2022). These dynamic interactions help ensure that NO is available when needed, particularly in response to conditions like hypoxia and exercise, where vasodilation and increased tissue oxygen delivery are critical (Kim-Shapiro et al., 2005; Umbrello et al., 2013). In this context, it is worth noting that myoglobin is expressed in VSMCs and contributes to nitrite-dependent generation of NO in response to hypoxia independently of eNOS or iNOS (Totzeck et al., 2012).

### Regulation of eNOS by substrates, co-factors, and product recycling

**FIGURE 5:**
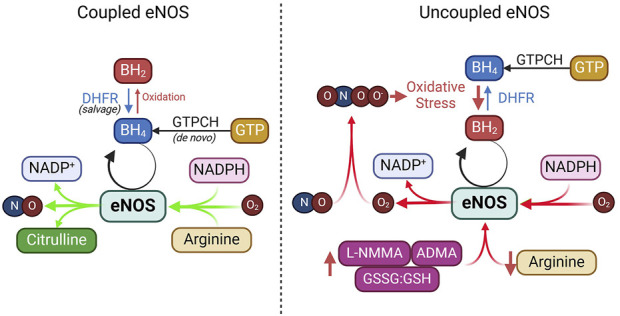
Coupled vs. uncoupled eNOS. See text for details. ADMA, asymmetric dimethylarginine; DHFR, dihydrofolate reductase; GTPCH, GTP cyclohydrolase I; GSSG, glutathione disulfide; GSH, glutathione; L-NMMA, NG-Monomethyl-L-arginine; NADPH, nicotinamide adenine dinucleotide phosphate.

The availability of substrates, co-factors, and the efficiency of product recycling are critical factors that dictate the outcome of eNOS activation. Disruptions in substrate availability, co-factor balance, or recycling processes—due to oxidative stress, metabolic disorders, or nutrient deficiencies—can impair eNOS activity and reduce NO bioavailability (Figure 5).

*L-arginine* is the primary substrate for eNOS and is converted into NO and L-citrulline during the enzymatic reaction. Given its high affinity for eNOS (K_*m*_ ∼2–3 μM) and an intracellular concentration above 100 μM, L-arginine is considered abundant in endothelial cells (Bode-Boger et al., 2007; Hardy and May, 2002). Nevertheless, extracellular L-arginine at millimolar concentrations can stimulate NO production (McDonald et al., 1997a; McDonald et al., 1997b). This has been considered the “arginine paradox.” Several mechanisms have been proposed to explain this phenomenon. One hypothesis involves the close association of eNOS with arginine transporters like CAT-1 and L-arginine recycling enzymes such as argininosuccinate lyase (ASL) (Erez et al., 2011). Other factors like competition from other amino acids, reduced uptake by cationic amino acid transporters (CATs), and elevated arginase activity—common in conditions such as hypertension and diabetes—can limit L-arginine availability to eNOS, diverting it away from NO production (McDonald et al., 1997a; McDonald et al., 1997b). Additionally, asymmetric dimethylarginine (ADMA), an endogenous inhibitor of eNOS, competes with L-arginine for binding, reducing NO synthesis (Boger et al., 1998a; Boger et al., 1998b). In addition to reducing NO synthesis, ADMA removes the suppression by NO of T-type voltage-dependent Ca^2+^ channels in VSMCs (Smith et al., 2020), triggering depolarizing Ca^2+^ spikes in VSMCs leading to vasoconstriction (Ng et al., 2024).

*Tetrahydrobiopterin (BH*_*4*_ *)* is an essential cofactor for eNOS required for efficient electron transfer during the synthase’s catalytic cycle (Alp and Channon, 2004; Crabtree et al., 2009a; Bendall et al., 2014). BH_4_ keeps eNOS in a “coupled” state, in which it synthesizes NO rather than harmful superoxide (Alp and Channon, 2004; Crabtree et al., 2009b). BH_4_ can be produced through two pathways: *de novo synthesis*, regulated by the rate-limiting enzyme guanosine triphosphate cyclohydrolase I (GTPCH), or through *the salvage pathway*, where dihydrobiopterin (BH_2_) is recycled back to BH_4_ by dihydrofolate reductase (DHFR) (Crabtree et al., 2009b; Crabtree and Channon, 2011). Under oxidative stress, BH_4_ is rapidly oxidized into BH_2_ by superoxide anions or peroxynitrite, which is particularly strong during NO scavenging (Milstien and Katusic, 1999). This oxidative depletion of BH_4_ causes uncoupling of eNOS, where it produces superoxide instead of NO.

*NADPH* is a critical electron donor in eNOS and facilitates electron transfer through FAD and FMN for effective NO production. It also maintains the redox state of BH_4_, ensuring efficient NO synthesis (Reyes et al., 2015). Depletion of NADPH, as seen in glucose-6-phosphate dehydrogenase deficiency or due to excessive consumption by NADPH oxidases, leads to eNOS uncoupling and oxidative stress (Noreng et al., 2022). Additionally, activation of CD38 in post-ischemic heart injury can severely deplete NADPH, impairing eNOS function and disrupting NADPH-dependent BH_4_ salvage and synthesis (Reyes et al., 2015).

*FAD and FMN*, both derived from riboflavin (vitamin B_2_), are essential co-factors for eNOS’s electron transfer process. These flavins facilitate the flow of electrons from NADPH to the oxygenase domain, ensuring efficient NO production (Figure 1). Deficiency in FAD and FMN due to poor dietary intake or metabolic disorders can impair eNOS activity, resulting in reduced NO synthesis and increased oxidative stress.

### eNOS uncoupling

Under certain conditions, eNOS can become “uncoupled,” shifting from producing NO to generating superoxide (O_2_⋅⁻), which not only reduces NO availability but also increases oxidative stress, reducing endothelial dysfunction. This section briefly explores the main causes of eNOS uncoupling and their therapeutic implications (Figure 5).

#### BH_4_ deficiency

Suboptimal levels of BH_4_, or a decreased BH_4_/BH_2_ ratio, are significant contributors to eNOS uncoupling observed in hypertension, diabetes, and atherosclerosis (Vasquez-Vivar et al., 1998; Wever et al., 1997; Soltis and Cassis, 1991; Hong et al., 2001; Landmesser et al., 2003; Lee et al., 2009). Studies in animal models, such as hypertensive mice and rats and apolipoprotein E-deficient mice, have demonstrated that oxidative depletion of BH_4_ leads to increased eNOS-derived superoxide, which impairs vasorelaxation (Hong et al., 2001; Landmesser et al., 2003; Laursen et al., 2001). In humans, decreased BH_4_ levels and eNOS uncoupling have been linked to coronary artery disease, diabetes, and hypertension (Antoniades et al., 2007; Ismaeel et al., 2020; Heitzer et al., 2000b; Stroes et al., 1997). This imbalance is further exacerbated by oxidative stress, which directly oxidizes BH_4_ to BH_2_, reducing BH_4_ availability for NO synthesis (Vasquez-Vivar et al., 1998; Laursen et al., 2001; Guzik et al., 2002; Alp et al., 2003; Bendall et al., 2005). Additionally, reduced expression of GTP-cyclohydrolase 1, the rate-limiting enzyme in BH_4_ synthesis, and dihydrofolate reductase, the enzyme that recycles BH_2_ to BH_4_, further contributes to eNOS uncoupling in diabetes and hypercholesterolemia (Alp and Channon, 2004; Xu et al., 2009; Xu J. et al., 2007; Whitsett et al., 2007; Munzel and Daiber, 2017; Chuaiphichai et al., 2017).

#### L-arginine deficiency or imbalance

L-arginine depletion is a significant contributor to eNOS uncoupling. Although intracellular L-arginine concentration typically far exceeds its K_*m*_ for eNOS, obesity, diabetes, and cardiovascular diseases can cause L-arginine deficiency. In such cases, reduced availability of L-arginine limits its ability to act as a substrate for eNOS, favoring the production of ROS over that of NO (Bode-Boger et al., 2007; Yang and Ming, 2013). A key mechanism of L-arginine depletion is upregulation of arginase, an enzyme that competes with eNOS for L-arginine and converts it into urea and L-ornithine. Arginase induction is particularly prevalent in obesity, diabetes, and atherosclerosis (Lass et al., 2002). For example, oxidized LDL significantly increases ARG2 expression levels in ECs (∼20%) but increases its activity by ∼80% (Ryoo et al., 2006). Many other factors can upregulate the expression level and activity of arginase, such as lipopolysaccharide, TNF-α, glucose, thrombin, hypoxia and angiotensin II [reviewed in Pernow and Jung (2013)]. Inhibiting arginase or supplementing L-arginine can restore NO production and improve endothelial function in these conditions (Chicoine et al., 2004). Furthermore, imbalance between L-arginine and ADMA, an endogenous inhibitor of eNOS, leading to reduced L-arginine/ADMA ratio, contributes to eNOS uncoupling (Closs et al., 2012; Watson et al., 2016; Peyton et al., 2018).

#### Oxidative stress and reactive oxygen species (ROS)

Oxidative stress plays a pivotal role in disrupting endothelial function by promoting eNOS uncoupling. Excessive ROS overwhelms the antioxidant defense system and causes oxidative stress that damages lipids, proteins, and DNA, contributing to the development of CVD (Thannickal and Fanburg, 2000; Costa et al., 2021; Sharifi-Rad et al., 2020; Li H. et al., 2013). Risk factors such as dyslipidemia, diabetes, hypertension, obesity, and smoking increase ROS levels, increasing endothelial dysfunction and CVD progression (Cai and Harrison, 2000; Xiang et al., 2021; Jurcau and Ardelean, 2022). ROS also play a key role in ischemia-reperfusion injury (Xiang et al., 2021; Jurcau and Ardelean, 2022). In the vascular wall, enzymes such as NADPH oxidase, xanthine oxidase, and uncoupled eNOS generate ROS, particularly superoxide. Superoxide reacts with NO to form peroxynitrite (ONOO^−^), a toxic compound that depletes NO and worsens endothelial dysfunction (Li H. et al., 2014; Thomson et al., 1995; Victor et al., 2009). NADPH oxidases, particularly NOX_2_, are major sources of superoxide in diabetes, hypertension, smoking, and aging (Konior et al., 2014; Gray et al., 2013; Manea et al., 2018; Fukui et al., 1997; Marchi et al., 2016; Kim et al., 2014; Jiang et al., 2011). The oxidative depletion of BH_4_ by ROS, especially via NADPH oxidases, further exacerbates eNOS uncoupling (Konior et al., 2014; Zhang et al., 2020).

#### eNOS glutathionylation

S-glutathionylation at Cys689 and Cys908 in the reductase domain results in eNOS uncoupling (Figure 3) (Chen et al., 2010; Chen et al., 2013; Suvorava et al., 2015; Zweier et al., 2011). This modification typically occurs under oxidative stress, when the ratio of reduced glutathione (GSH) to oxidized glutathione (GSSG) decreases (Suvorava et al., 2017; Benson et al., 2013; Daiber et al., 2002). S-glutathionylation disrupts the alignment of FAD and FMN, essential cofactors for eNOS’s electron transport, resulting in superoxide production instead of NO (Chen et al., 2010; Zweier et al., 2011). This mechanism of eNOS uncoupling is unique in that it occurs in the reductase domain, unlike other uncoupling mechanisms that occur in the oxygenase domain, and can be inhibited by L-NAME (Chen et al., 2010). *In vivo* studies have confirmed the association of eNOS S-glutathionylation with endothelial dysfunction in hypertension, aging, and cardiovascular diseases (Chen et al., 2010; Suvorava et al., 2015). In *ex vivo* rat aortic segments and spontaneously hypertensive rats, high levels of eNOS S-glutathionylation correspond with impaired vasodilation. S-glutathionylation can be reversed by Grx-1 (Chen et al., 2013; Shang et al., 2017).

## Strategies to enhance endogenous no production

The use of exogenous NO donors is limited by the short-lived nature of their effects and the development of nitrate tolerance. Enhancing endogenous NO production should provide physiological and more sustained effects. Current strategies involve promoting eNOS activity by providing essential substrates or cofactors or by pharmacologically upregulating eNOS expression and activity; this could also be done by preventing/reversing eNOS uncoupling. We will divide our review below of these approaches based on the level of available evidence.

### Approaches with both preclinical and clinical evidence

#### L-arginine supplementation

##### Rationale

**FIGURE 6:**
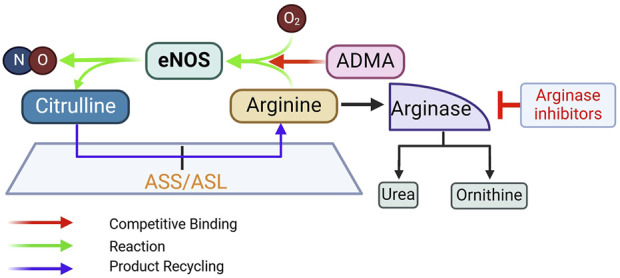
Rationale for supplementing L-arginine, inhibiting arginase, and supplementing L-citrulline to promote eNOS activity. ADMA, asymmetric dimethylarginine; ASL, argininosuccinate lyase; ASS, argininosuccinate synthase. See text for more details.

L-arginine supplementation to promote NO production is based on the premise that in conditions of endothelial dysfunction, intracellular L-arginine levels may become suboptimal. It is also based on the “arginine paradox,” discussed above, that despite an intracellular L-arginine concentration much higher than its K_*m*_ for eNOS activity, high extracellular concentration of L-arginine can still promote NO production. Additionally, L-arginine also competes with ADMA, an endogenous eNOS inhibitor (Boger et al., 1998c), thereby restoring the L-arginine/ADMA ratio that is critical for eNOS activity (Dong et al., 2011) (Figure 6).

##### Evidence

Early preclinical studies provided evidence that oral L-arginine supplementation improves acetylcholine (ACh)-induced vasorelaxation and NO production (Girerd et al., 1990; Cooke et al., 1991; Rossitch et al., 1991; Boger et al., 1995). Clinical studies began with *intravascular injection of L-arginine* in small groups of subjects. In hypercholesterolemic patients (n = 8, mean age 51.5) with slight luminal irregularities of the left anterior descending coronary artery (LAD), ACh-induced reduction in coronary blood flow, an indication of endothelial dysfunction, is improved by intracoronary injection of L-arginine (Drexler et al., 1991). In patients with diffuse atherosclerotic LADs (n = 13, age 56 ± 7.5), direct intracoronary L-arginine injection also ameliorates the ACh-induced vasoconstriction and reduction in blood flow (Dubois-Rande et al., 1992). In patients with critical peripheral limb ischemia (n = 10, age 68.3 ± 3.1), a single intravenous dose of L-arginine significantly increases blood flow, accompanied by urinary cGMP excretion (Bode-Boger et al., 1996). *Overall*, *intravascular L-arginine delivery in humans offers acute improvement of endothelial function*. In young healthy subjects (n = 80, age 25.4 ± 0.2), this effect is achieved with higher doses and is correlated with L-arginine plasma concentrations (Bode-Boger et al., 1998).

From clinical trials testing the effects of oral administration of L-arginine, a picture has emerged that *short-term L-arginine administration (≤3 months) provides benefits, whereas longer supplementation (≥6 months) gives mixed results*. For example, 4-week L-arginine supplementation improves reactive hyperemia in patients with hypertension and hyperhomocysteinemia (2.4 g/d, n = 25, age 40–65) (Reule et al., 2017); and flow-mediated dilation is improved by 3-month L-arginine supplementation in hypertensive subjects (2.4 g/d, n = 40, age 40–65) (Menzel et al., 2018). In patients with a history of coronary bypass surgery, 6-month supplementation with L-arginine (6.4 g/d, n = 32, age 65 ± 10) reduces ADMA levels, increases plasma cGMP, and improves reactive hyperemia compared to placebo (n = 32, age 64 ± 11) (Lucotti et al., 2009). However, 6-month supplementation with L-arginine in patients with peripheral arterial disease (3 g/d, n = 66, age 73 ± 9) does not increase NO synthesis or improve vascular reactivity vs. placebo (3 g/d, n = 67, age 72 ± 7) (Wilson et al., 2007) or alter vascular stiffness in post-myocardial infarction (MI) patients (3 × 3 g/day, n = 75, age 60.4 ± 12.9) (Schulman et al., 2006). This has led to the “*arginine tolerance*” hypothesis (Wilson et al., 2007), akin to the common nitrate tolerance phenomenon.

##### Challenges

Given the state of current evidence, major cardiovascular guidelines do not include L-arginine supplementation for the prevention of CVD. Among explanations for the absence of effect of long-term L-arginine administration in some clinical trials, oxidative depletion of BH_4_ can result in eNOS uncoupling even with sufficient L-arginine supply (Xiong et al., 2014; Scalera et al., 2009). L-arginine also increases arginase expression, which in turn reduces L-arginine availability (Caldwell et al., 2018; Wang et al., 2006), a mechanism that may partly explain the arginine tolerance hypothesis. Nevertheless, interpretation of results of clinical trials for oral L-arginine should include whether the treatment regimens include other drugs that also promote eNOS function and whether there is baseline L-arginine deficiency. For example, in post-MI patients (n = 78, age 60.2 ± 14.2), addition of L-arginine (3 × 3 g/d) to the post-MI treatment regimen for 6 months does not improve vascular stiffness or left ventricular function compared to patients receiving placebo (n = 75, age 60.4 ± 12.9) (Schulman et al., 2006). However, in addition to the normal baseline L-arginine levels in both groups, this treatment regimen includes aspirin, which acetylates eNOS and promotes NO production (Taubert et al., 2004); clopidogrel, which stimulates eNOS phosphorylation (Schafer et al., 2011); an ACE inhibitor, which inhibits degradation of bradykinin, thereby triggering endothelial Ca^2+^ signals and activating eNOS (Bachetti et al., 2001); and a statin, which stabilizes eNOS mRNA and activates the PI3K/Akt pathway to activate the synthase (Wang et al., 2005). Similarly, in patients with hypertension and hyperhomocysteinemia (18 males and 7 females, age 40–65), reactive hyperemia is improved by 4 weeks oral L-arginine (Reule et al., 2017), administered in a mixture with pycnogenol, which itself activates eNOS (Fitzpatrick et al., 1998); α lipoic acid, which promotes eNOS recoupling (Sena et al., 2008); vitamin B2, which is a cofactor for eNOS; and folic acid, which activates eNOS and prevents its uncoupling (Stroes et al., 2000). The effects of co-treatment agents on eNOS may obscure or reduce the observed effect of L-arginine on endothelial function.

#### Inhibiting arginase

##### Rationale

Arginase was identified in 1904 as the enzyme that hydrolyzes L-arginine into ornithine and urea (Kossel and Dakin, 1904). Two isoforms exist – *Arg1* and *Arg2*. *Arg1* is expressed abundantly in hepatic tissue, where it plays a major role in the urea cycle, whereas *Arg2* is more predominant in extrahepatic tissues. Both isoforms are expressed in ECs (Buga et al., 1996); and many risk factors of CVD can increase the expression levels and activity of endothelial arginase (Pernow and Jung, 2013; Ryoo et al., 2008). Thus, arginase inhibition in ECs is predicted to increase L-arginine availability for NO production by eNOS (Figure 6).

##### Evidence

Considering a high K_*m*_ value for L-arginine as substrate for arginase (1–3 mM), it has been reasoned that the main function of arginase is to limit the intracellular accumulation of L-arginine; whereas given a low K_*m*_ value for L-arginine as substrate of NOS (10–20 µM), arginase may not affect basal NO production, unless L-arginine level falls below 10–20 µM (Buga et al., 1996). Nevertheless, the Vmax for L-arginine of arginase is ∼1,000-fold higher than that of the NO synthases (Wu and Morris, 1998). So theoretically, arginase can compete with eNOS for L-arginine and affect NO synthesis. Indeed, treatment of ECs with L-arginine increases production of both urea and NO, while treatment with L-valine, which inhibits arginase [IC_50_ ∼ 6.2 ± 0.4 mM (Paik et al., 1978)], increases NO production only; consistently, combined treatment with L-arginine and L-valine shifts the balance towards NO synthesis over urea formation (Chicoine et al., 2004). Additionally, overexpression of both arginase isoforms reduces L-arginine availability (Li et al., 2001). Many arginase inhibitors with high affinity have been developed over the past several decades, such as N-hydroxy-L-arginine [NOHA, K_*i*_ = 3.6 µM for human ARG1 (Di Costanzo et al., 2010), 1.6 µM for human ARG2 (Colleluori and Ash, 2001)], N-hydroxy-nor-L-arginine [nor-NOHA, K_*d*_ = 0.47 µM for human ARG1 (Di Costanzo et al., 2010), 51 nM for human ARG2 (Colleluori and Ash, 2001)] and boronic acid derivatives like 2(S)-amino-6-hexanoic acid (ABH) [IC_*50*_ values of derivatives range 17–1,470 nM for human ARG1, 30–2,150 nM for human ARG2 (Collet et al., 2000; Ilies et al., 2011)] and S-(2-boronoethyl)-l-cysteine (BEC) [K_*i*_ = 0.4–0.6 µM (Busnel et al., 2005)]. A comprehensive review of arginase inhibitors can be found in Pudlo et al. (2017). In *preclinical models,* arginase inhibition prevents eNOS uncoupling and atherogenesis. In hypercholesterolemic mice, deletion of *Arg2* or inhibition of arginase with BEC improves endothelial function and reduces atherosclerosis (Ryoo et al., 2008). Similarly, in diet-induced obesity models, arginase inhibition with ABH or deletion of *Arg1* or *Arg2* reverses vascular dysfunction (Bhatta et al., 2017; Atawia et al., 2019). In diabetic mice, the use of ABH effectively restores endothelial function (Pernow et al., 2015). *Clinical studies* in small groups of patients have shown effect of *intravascular* injection of nor-NOHA in several vascular beds in various conditions of EC dysfunction. Intra-arterial infusion of nor-NOHA (0.1 mg/min) improves endothelium-dependent vasorelaxation in radial artery following ischemia-reperfusion injury in male patients (average age 65) with coronary artery disease (CAD, n = 12) or CAD and type 2 diabetes (T2D, n = 12) (Kovamees et al., 2014). The same dose of nor-NOHA administration also improves endothelium-dependent vasorelaxation in forearm vessels in patients with heterozygous familial hypercholesterolemia (n = 12, age 32) and healthy controls (n = 12, age 30) (Kovamees et al., 2016a). Similarly, 0.1 mg/min infusion of nor-NOHA improves endothelium-dependent vasodilation in the forearm microvasculature in T2D patients (n = 12, age 66 ± 4), but not in age-matched healthy subjects (n = 12) (Kovamees et al., 2016b). Intracoronary infusion of nor-NOHA (0.1 mg/min) also enhances endothelium-dependent vasorelaxation in coronary arteries in patients (age 69 ± 3) with CAD (n = 16), or CAD and T2D (n = 16), but not in healthy subjects (n = 16) (Shemyakin et al., 2012). These effects appear to be more pronounced in aged than in younger subjects (n = 21, age 48–75) (Mahdi et al., 2019), and does not depend on efficacy of blood glucose-lowering therapy in T2D patients (n = 16, average age 64) (Mahdi et al., 2018). Nor-NOHA improves EC dysfunction in T2D patients (n = 26), but not in type 1 diabetes (T1D) patients (n = 13) with equal blood glucose levels (Tengbom et al., 2024).

##### Challenges

Clinical trials testing the effect of *oral arginase inhibitors* on endothelial function are currently not available, due perhaps to the fact that most oral formulations of arginase inhibitors have poor oral bioavailability (Pudlo et al., 2017). Some clinical trials have begun to test new oral arginase inhibitors in cancer therapy, based on the high expression level of ARG1 in immunosuppressive cells. Examples include OATD-02, a recently developed ARG1/2 dual inhibitor with ∼15 nM affinity (Borek et al., 2023) and CB-1158, which has IC_*50*_ values of 86 nM and 296 nM for ARG1 and ARG2, respectively (Naing et al., 2024; Steggerda et al., 2017). However, using oral arginase inhibitors long-term to promote NO production may be challenged by the lack of isoform specificity, making it difficult to balance the roles of arginase isoforms in the urea cycle and NO production and potential disruption of nitrogen metabolism (Pudlo et al., 2017). While nor-NOHA is specific for ARG2, it is not available in oral formulations due to issues with stability and absorption with oral administration. Overall, due to the lack of clinical trials, arginase inhibitors are not currently recommended for the prevention or treatment of cardiovascular disease in clinical practice guidelines.

#### L-citrulline supplementation

##### Rationale

**FIGURE 7:**
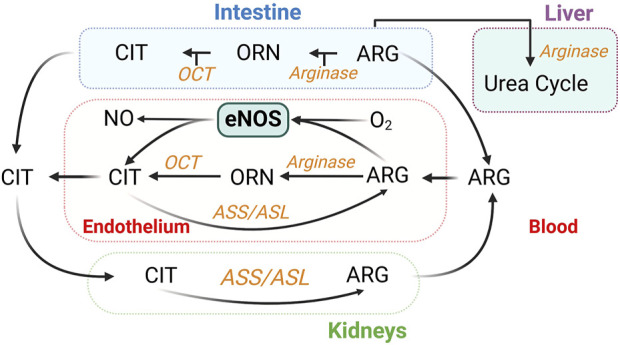
Inter-organ metabolism of L-citrulline (CIT) and L-arginine (ARG). Endothelium-derived NO synthesis takes place in all organs. Urea is also generated in the kidneys (Levillain, 2012). ASL, argininosuccinate lyase; ASS, argininosuccinate synthase; OCT, ornithine transcarbamylase; ORN, ornithine.

Oral L-arginine undergoes first-pass metabolism [∼38% in humans (Castillo et al., 1993)], whereas L-citrulline is converted into L-arginine via the argininosuccinate pathway, in which argininosuccinate synthase (ASS) catalyzes the formation of argininosuccinate from citrulline and aspartate; argininosuccinate is subsequently cleaved to form arginine and fumarate by ASL. Citrulline conversion to arginine takes place in multiple cell types, including ECs (Hecker et al., 1990; Wu and Meininger, 1993) (Figure 7). Oral L-citrulline has high intestinal absorption but minimal first-pass metabolism and renal reabsorption (Bahadoran et al., 2021), yielding more stable increases in plasma L-arginine levels. L-citrulline is also an inhibitor of arginase activity, as measured by the production of urea (Shearer et al., 1997). L-citrulline supplementation is thus a promising way to promote NO production (Yu et al., 1997; Agarwal et al., 2017; Osowska et al., 2004).

##### Evidence

L-citrulline administration as a means to promote NO production has received significant attention recently. In *cell models* such as ECs exposed to hypoxia or high glucose, L-citrulline supplementation restores NO bioavailability and reduces ARG2 expression level and activity (Douglass et al., 2023; Tsuboi et al., 2018). In *animal studies*, L-citrulline increases NO production and improves vascular function, with positive effects on glucose tolerance, exercise capacity, and placental angiogenesis (Li et al., 2023; Eshreif et al., 2020). Several *clinical studies* in the past decade have examined the effects of L-citrulline administration in small cohorts with endothelial dysfunction. In patients with vasospastic angina (n = 22, age 41–64), L-citrulline supplementation (0.8 g/d, 4–8 weeks) increases brachial artery flow-mediated dilation, plasma NOx levels, and L-arginine/ADMA ratio (Morita et al., 2013). In T2D patients (n = 25, age 25–65), consumption of 1 g L-citrulline twice a day for 4 weeks reduces the T2D-induced increase in arginase activity and increases plasma nitrites, effects corroborated by improvement of ACh-induced relaxation in *ex vivo* mouse aortic rings incubated (24-h) in low or high glucose-containing media and increases in NO production in ECs treated with high glucose (Shatanawi et al., 2020). In hypertensive postmenopausal women (n = 14, age 61 ± 6), high-dose L-citrulline (10 g/d, 4 weeks) improves flow-mediated dilatation and aortic stiffness and reduces blood pressure (Maharaj et al., 2022). Most recently, in hypertensive postmenopausal women (n = 14, age 60 ± 1), L-citrulline (10 g/d, 4 weeks) reduces the increases in systolic blood pressure and pulse pressure induced by isometric handgrip exercise and the pressures of forward and backward aortic waves during postexercise muscle ischemia (Dillon et al., 2024).

##### Challenges

Positive effects have been demonstrated of drastically different daily doses of L-citrulline in recent studies, from 0.8 g/d (Morita et al., 2013) to 10 g/d (Maharaj et al., 2022; Dillon et al., 2024). While side effects have not been reported, L-citrulline exerts a dose-dependent effect to activate arginase activity in a cell-based study (Douglass et al., 2023). This may limit its own effects via reduction in L-arginine availability. In addition, high doses of L-citrulline might also affect nitrogen metabolism, based on its complicated interorgan metabolism (Breuillard et al., 2015) (Figure 7). While there are promising results, more large-scale, long-term clinical trials are needed to establish the role of L-citrulline in the prevention or treatment of CVD.

#### Folic acid supplementation

##### Rationale

**FIGURE 8:**
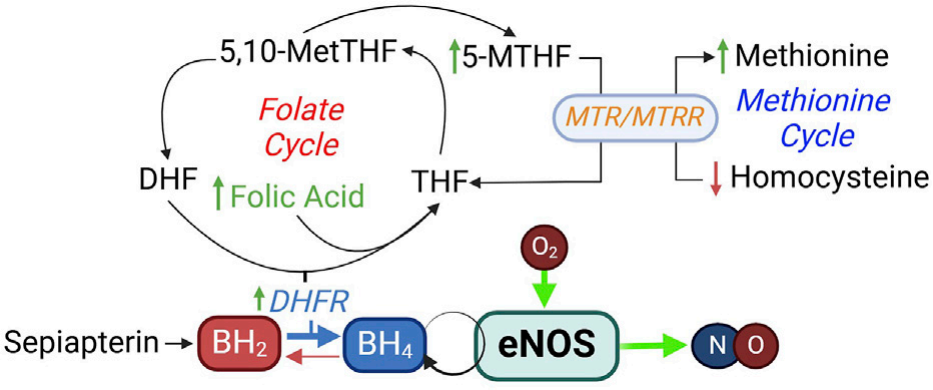
Folic acid in BH_4_ synthesis and homocysteine metabolism. DHF, dihydrofolate; DHFR, dihydrofolate reductase; MTHF, methylhydrofolate; MTR, methionine synthase; MTRR, methionine synthase reductase.

Folic acid cannot be synthesized by the body and can only come from diet or supplementation. In addition to important role in the folate cycle that is involved in the reduction of BH_2_ to BH_4_, it is an important component in homocysteine metabolism via the methionine cycle (Blom and Smulders, 2011) (Figure 8), and is critical for neural tube development. Increases in plasma homocysteine concentration is strongly associated with cardiovascular disease, and supplementation of folic acid can lower plasma homocysteine levels (Kaye et al., 2020). Folic acid supplementation appears to have a variety of beneficial cardiovascular effects. These include reducing oxidative stress and inflammation, improving plasma lipid profile, and control of blood pressure and blood glucose levels (Asbaghi et al., 2021a; Asbaghi et al., 2021b; Asbaghi et al., 2022; Asbaghi et al., 2021c; Asbaghi et al., 2023).

##### Evidence

Studies *in cells* and *animals* have supported a direct role of folic acid to enhance NO availability via effects on eNOS and DHFR expression and activity (Stroes et al., 2000; Gao et al., 2009). In regard to eNOS function, 5-methyltetrahydrofolate (MTHF), the active form of folic acid, does not have an effect on pterin-free eNOS, but directly interferes with BH_4_-repleted, partially uncoupled eNOS, shifting it from superoxide production toward NO production (Stroes et al., 2000). Folic acid enhances DHFR expression and activity, thereby increasing BH_4_ and NO availability and reducing oxidant production in ECs and in mice (Gao et al., 2009). Some small-scale *clinical trials* (sample sizes ∼16–50 participants) have indicated improvement of endothelial function with folic acid supplementation. A recent meta-analysis of 21 randomized controlled trials on the effect of folic acid supplementation on endothelial function suggests that this intervention significantly improves flow-mediated dilation percentage (FMD%) and flow-mediated dilation, but not end-diastolic diameter (EDD) or intracellular adhesion molecule 1 (ICAM-1) expression (Zamani et al., 2023).

##### Challenges

Due to its well-established role in neural tube development, folic acid supplementation is recommended in clinical guidelines (US Preventive Services Task Force, American College of Obstetricians and Gynecologists, American Academy of Family Physicians, American Academy of Pediatrics, and the Center for Disease Control and Prevention) for those who are planning to or could become pregnant (Force et al., 2023). However, given the lack of large-scale trials, folic acid supplementation is currently not recommended for cardiovascular disease prevention or treatment, although it may be considered in specific populations, such as those with elevated homocysteine levels (Kaye et al., 2020) or in regions with low dietary folate intake.

#### Increasing BH_4_ supply

##### Rationale

As reviewed above, BH_4_ deficiency is a common cause of eNOS uncoupling in many cardiovascular diseases (Figure 5) (Vasquez-Vivar et al., 1998; Wever et al., 1997; Soltis and Cassis, 1991; Hong et al., 2001; Landmesser et al., 2003; Lee et al., 2009). Sapropterin is a synthetic formulation of BH_4_. Sepiapterin is a stable precursor in the salvage pathway of BH_4_ biosynthesis, which converts sepiapterin to BH_4_ via the activities of sepiapterin reductase and DHFR (Figure 8). It has higher cell permeability than BH_4_ (Sawabe et al., 2008).

##### Evidence

Many *preclinical studies* have examined effects of BH_4_ or sepiapterin administration on NO-dependent functions in the cardiovascular system. For example, BH_4_ administration following transverse aortic constriction (TAC) for 5 weeks in mice recouples eNOS and reduces oxidative stress, reverses cardiac hypertrophy and fibrosis, and improves cardiac function (Moens et al., 2008). Similarly, oral administration of sepiapterin for 8 weeks after TAC increases NO availability, inhibits oxidative stress, and reduces cardiomyocyte hypertrophy (Yoshioka et al., 2015). Of note, in the model of myocardial infarction, iNOS expression is upregulated, and oral sepiapterin administration inhibits nitrotyrosine formation and increases in nitrite and nitrate in wildtype, eNOS^−/−^, and nNOS^−/−^ mice, but not iNOS^−/−^ mice; these effects are associated with prevention of cardiac remodeling and dysfunction (Shimazu et al., 2011). Similar findings are observed in an MI model with streptozotocin-induced T2D (Jo et al., 2011). Multiple small-scale *clinical studies* (5–30 participants each) have shown BH_4_ supplementation can improve endothelial function in various cardiovascular conditions. Most early studies tested the effects of *intravascular delivery of BH*_*4*_. These studies showed that BH_4_ injection improves forearm blood flow in patients with hypercholesterolemia (Stroes et al., 1997), hypertension (Higashi et al., 2002) and T2D (Heitzer et al., 2000b). BH_4_ injection also increases coronary endothelial function in patients with CAD (Maier et al., 2000; Setoguchi et al., 2001; Fukuda et al., 2002), and increases skin blood flow responses to local heating, an indication of microvasculature EC function, in hypercholesterolemic patients (Stroes et al., 1997; Holowatz and Kenney, 2011). Nevertheless, in some studies, intraarterial infusion of BH_4_ fails to improve endothelial function (Nystrom et al., 2004; Worthley et al., 2007). *Chronic oral supplementation* of BH_4_ has only been examined in limited number of trials. In patients with poorly controlled hypertension (n = 16, age 57 ± 9), 400 mg/d oral BH_4_ for 4 weeks significantly improves brachial flow-mediated vasodilation and reduces BP (Porkert et al., 2008). In hypercholesterolemic patients (n = 22, age 60.8 ± 9.2), oral BH_4_ (400 mg bid) for 4 weeks normalizes ACh-induced vasodilation, vascular oxidative stress, and NO and superoxide production (Cosentino et al., 2008).

##### Challenges

Despite evidence that BH_4_ and its analogs can improve endothelial function, the lack of large-scale trials and mixed outcomes of the published small-scale clinical studies have prevented their translation into current practice guidelines for CVD prevention and management (Heidenreich et al., 2022). Additionally, issues such as the oxidation of BH_4_ to inactive forms like BH_2_ limit its therapeutic potential (Cunnington et al., 2012). Of note, BH_4_ is also a cofactor of phenylalanine hydroxylase, which converts phenylalanine to tyrosine, a precursor to catecholamines. Large-scale trials are only available for BH_4_, sepiapterin or sapropterin in phenylketonuria (Muntau et al., 2024), an autosomal recessive disorder caused by deficiency of phenylalanine hydroxylase, leading to hyperphenylalaninemia, developmental delay, behavioral problems, and reduced quality of life (Blau et al., 2010).

### Approaches with preclinical evidence only

#### Promoting transcriptional regulation of eNOS

Several compounds have been identified to enhance eNOS transcription and promote NO production. *AVE3085* is a novel synthetic agent that acts as an eNOS transcription enhancer. Oral administration of AVE3085 for 7 days increases eNOS expression and activity, reverse eNOS uncoupling, reduces oxidative stress and improve endothelial function in diabetic mice (Cheang et al., 2011). Four-week administration of AVE3085 upregulates eNOS mRNA and protein levels, enhances eNOS phosphorylation and decreases nitrotyrosine formation, leading to improved endothelium-dependent vasorelaxation in hypertensive animals (Yang et al., 2011). These effects are absent in eNOS^−/−^ mice (Cheang et al., 2011; Yang et al., 2011). *AVE9488* is another eNOS transcription enhancer that has been shown to increase eNOS expression and activity, reverse eNOS uncoupling, protect against ischemia-reperfusion cardiac injury, and improve post-infarct function (Sasaki et al., 2006; Wohlfart et al., 2008; Frantz et al., 2009; Fraccarollo et al., 2008). More recently, *LEENE,* a long non-coding RNA induced downstream of KLF2/KLF4 has been shown to form proximity association with the eNOS promoter and enhances eNOS transcription through chromatin association (Miao et al., 2018). Atorvastatin increases LEENE expression, thereby upregulating eNOS expression, while inhibition of LEENE strongly enhances ECs-monocyte adhesion induced by pulsatile shear stress (Miao et al., 2018). No clinical trials have examined the effects of these transcription enhancers on endothelial function in humans.

#### Modulating eNOS localization

Disrupting the inhibitory interaction between Cav-1 and eNOS (Figure 4) should promote eNOS activation and NO production. Cavnoxin, a 20-amino-acid peptide designed based on the caveolin scaffolding domain with alanine substitutions at key inhibitory residues (T90, T91, F92) (Bernatchez et al., 2005), releases eNOS from inhibition. In wild-type mice, cavnoxin administration significantly increases NO levels, reduces mean blood pressure, and improves vascular function, effects that are lost in eNOS^−/−^ and Cav-1^−/−^ mice (Bernatchez et al., 2011). Additionally, chronic cavnoxin treatment reduced atherosclerosis in ApoE-knockout mice and diabetic atherosclerotic models.

#### Targeting Hbα

Disrupting Hbα-eNOS interaction can reduce the NO-scavenging action of Hbα (Figure 4). In addition, inhibiting CytB5R3 can increase NO availability by reducing Hb from its Fe^2+^ to Fe^3+^ form, thereby releasing NO from its high-affinity interaction. *HbαX*, a peptide based of the eNOS-interacting sequence of Hbα (a.a. 34–43), tagged to an HIV TAT cell-penetration sequence, has been developed to disrupt the interaction between Hbα and eNOS, which increases NO availability, acutely reducing blood pressure (Straub et al., 2014a). HbαX lowers blood pressure in mice with angiotensin II-induced hypertension and blunts vasoconstrictive response to phenylephrine in isolated human vessels (Keller et al., 2016). Pharmacological inhibition or genetic deletion of CytB5R3 increases NO availability in blood vessels (Straub et al., 2012).

#### Promoting dihydrofolate reductase activity

DHFR plays a critical role in regenerating BH_4_ from its oxidized form BH_2_ (Figure 5). Enhancing DHFR activity will help maintain or increase BH_4_ levels, thus preventing eNOS uncoupling and promoting NO production. Genetic knockdown of DHFR decreases BH_4_ levels and increases BH_2_ levels, uncoupling eNOS and reducing NO production (Crabtree et al., 2009b; Crabtree et al., 2011). Oxidative stress, such as that induced by angiotensin II, downregulates DHFR, exacerbating eNOS uncoupling and reducing NO availability (Chalupsky and Cai, 2005). In addition, DHFR S-nitrosylation by eNOS-derived NO stabilizes DHFR, thereby preventing its degradation and ensuring its activity in recycling BH_4_ (Cai et al., 2015). This highlights the interdependence between eNOS-derived NO and DHFR stability. *Challenges–*Due to the lack of specific DHFR activators, there are no clinical studies that examined the possibility of stimulating its activity to increase NO availability. Antimetabolic drugs such as methotrexate or fluorodeoxyuridine and hydroxyurea stimulate DHFR promoter (Eastman et al., 1991); however, their actions are non-specific.

## Conclusion and future perspectives

Nearly five decades have passed since NO was identified. Since eNOS was cloned (Marsden et al., 1992), our understanding of how NO availability is regulated has increased exponentially. Nevertheless, only general measures to improve eNOS function such as healthy lifestyle and physical exercise and approaches to amplify certain downstream components of NO signaling have made it to clinical practice guidelines. Among the salient indications, inhaled NO is used for acute pulmonary vasoreactivity testing and severe hypoxia in pulmonary arterial hypertension (PAH), nitrates are used extensively in ischemic heart disease and heart failure, sGC stimulators and activators are used for PAH, and PDE5 inhibitors are used for erectile dysfunction, PAH and benign prostatic hyperplasia.

This article has reviewed the basic regulatory mechanisms of eNOS activities and relevant approaches to improve endogenous NO production that have been tested to date but not yet approved clinically. We have highlighted the rationale, current evidence, and challenges that have prevented these approaches from entering the clinical armamentarium to improve endothelial function. Among the main challenges are the lack of specificity, lack of consistent efficacy of long-term treatment, and lack of large-scale clinical data.

Given this backdrop, what can be done? Obviously, *additional studies and large-scale trials* are needed in many cases. The lack of consistent outcomes of L-arginine supplementation could be due to multiple mechanisms, as discussed. Could *intermittent L-arginine delivery*, a strategy widely applied clinically to prevent nitrate tolerance to some degree, be considered and tested to reduce a potential “arginine tolerance”? In addition, because L-arginine supplementation increases arginase activity, which limits the efficacy, now that arginase inhibitors are available with better oral availability, *L-arginine supplementation could be considered in combination with arginase inhibition*. In regard to arginase inhibition, development of *ARG2-specific inhibitors* would help limit issues associated with disruption of the urea cycle in the liver, where ARG1 is more abundant. L-citrulline as a single supplementation appears to have yielded superior outcomes than L-arginine supplementation; however, large-scale trials are needed, and *identifying the minimum effective dose range for L-citrulline is necessary*, considering a potential effect of high doses on nitrogen balance. *For peptide-based interventions*, factors such as peptide stability and efficient delivery need to be addressed before clinical application can be considered.

*Biochemically*, it is worth noting that for NO-induced vasodilation, there does not need to be a high concentration of NO because of its picomolar affinity for sGC (Wu et al., 2022). Therefore, promoting NO production to a low level may preferentially promote vasodilation via the eNOS-NO-sGC pathway and prevent the formation of reactive nitrogen species. In addition, *targeting interventions specifically to the endothelium* is an ideal solution that is difficult to achieve but would reduce the required effective doses and limit many off-target effects. This would be particularly helpful for approaches aimed to target eNOS’s essential interacting partners and numerous transcriptional factors that are abundant also in other tissues.

## Glossary

**ABH**: 2(S)-amino-6-hexanoic acid
**ACh**: acetylcholine
**ADMA**: asymmetric dimethylarginine
**Akt**: protein kinase B
**AMPK**: AMP-activated protein kinase
**ASL**: argininosuccinate lyase
**ASS**: argininosuccinate synthase
**BEC**: S-(2-boronoethyl)-l-cysteine
**BH**_**2**_: dihydrobiopterin
**BH**_**4**_: tetrahydrobiopterin
**CAD**: Coronary Artery Disease
**CaM**: calmodulin
**CaMKII**: calcium/calmodulin-dependent protein kinase II
**CAT-1**: cationic amino acid transporter-1
**Cav-1**: caveolin-1
**cGMP**: cyclic GMP
**CSD**: caveolin-scaffolding domain
**CVD**: cardiovascular disease
**CytB5R3**: cytochrome b5 reductase 3
**DHFR**: dihydrofolate reductase
**Dyn2**: dynamin-2
**ECs**: endothelial cells
**EDD**: end-diastolic diameter
**eNOS**: endothelial nitric oxide synthase
**FAD**: flavin adenine dinucleotide
**FMD%**: flow-mediated dilation percentage
**FMN**: flavin mononucleotide
**GPCR**: G-protein coupled receptor
**Grx1**: glutaredoxin
**GSH**: reduced glutathione
**GSSG**: oxidized glutathione
**GTPCH**: guanosine triphosphate cyclohydrolase I
**Hbα**: alpha subunit of hemoglobin
HDL: high-density lipoprotein
HIF-1α: hypoxia-inducible factor-1 alpha
**Hsp90**: heat shock protein 90
**ICAM-1**: intracellular adhesion molecule 1
**iNOS**: inducible NOS
**KLF2/KLF4**: Krüppel-like factor 2/Krüppel-like factor 4
**LAD**: left anterior descending coronary artery
**lncRNAs**: long non-coding RNAs
**LPS**: lipopolysaccharide
**MEJ**: myoendothelial junctions
**MI**: myocardial infarction
**METHF**: 5-methyltetrahydrofolate
**NADPH**: nicotinamide adenine dinucleotide phosphate
**NF-κB**: nuclear factor kappa-light-chain-enhancer of activated B cells
**nNOS**: neuronal NOS
**NO**: nitric oxide
**NOHA**: N-hydroxy-L-arginine
**nor-NOHA**: N-hydroxy-nor-L-arginine
**NOS**: NO synthases
**NOSIP**: eNOS interacting protein
**NOSTRIN**: eNOS trafficking inducer
**NOX**_**2**_: NADPH oxidase 2
**Nrf2**: nuclear factor erythroid 2-related factor
**ox-LDL**: oxidized low-density lipoprotein
**PAH**: pulmonary arterial hypertension
**PDE**: phosphodiesterase
**PKA**: protein kinase A
**PKG**: protein kinase G
**PMCA**: plasma membrane Ca^2+^-ATPase
**PPIs**: protein-protein interactions
**PTMs**: post-translational modifications
**ROS**: reactive oxygen species
**sGC**: soluble guanylate cyclase
**SIRT1**: sirtuin 1
**SMAD2**: mothers against decapentaplegic homolog-2
**SNOs**: s-nitrosothiols
**SOCE**: store-operated Ca^2+^ entry
**Sp1**: specificity protein 1
**Sp3**: specificity protein 3
**STIM1**: stromal interaction molecule 1
**T1D**: type 1 diabetes
**T2D**: type 2 diabetes
**TAC**: transverse aortic constriction
**TGF-β**: tumor growth factor-beta
**TNF-α**: tumor necrosis factor-alpha
**VEGF**: vascular endothelial growth factor
**VSMCs**: vascular smooth muscle cells
